# Vitamin E-enriched medium cross-linked polyethylene in total knee arthroplasty (VIKEP): clinical outcome, oxidation profile, and wear analysis in comparison to standard polyethylene—study protocol for a randomized controlled trial

**DOI:** 10.1186/s13063-023-07811-1

**Published:** 2024-01-05

**Authors:** Kristin Maier, Marius Selig, Andréa Haddouche, Martin Haunschild, Oliver Hauschild, Iman Khalili, Julia Kirschberg, Christoph Lutter, Michael Menges, Patrice Mertl, Andreas Niemeier, Brice Rubens-Duval, Wolfram Mittelmeier

**Affiliations:** 1 Medical Scientific Affairs, Aesculap AG, Am Aesculap-Platz, 78532 Tuttlingen, Germany; 2 Hopital Sud – CHU Grenoble, Avenue Kimberley, 38130 Echirolles, France; 3 Klinik Für Allgemeine Orthopädie, Endoprothetik Und Kinderorthopädie, Katholisches Klinikum Koblenz-Montabaur, Kardinal-Krementz-Str. 1-5, Koblenz-Montabaur, 56073 Germany; 4 Department for Orthopedic and Trauma Surgery, Park-Klinik Weissensee, Schönstraße 80, Berlin, 13086 Germany; 5 Krankenhaus Reinbek St. Adolf-Stift, Hamburger Straße 41, 21465 Reinbek, Germany; 6 Waldkliniken Eisenberg, Klosterlausnitzer Straße 81, 07607 Eisenberg, Germany; 7 Orthopädische Klinik Und Poliklinik, Universitätsmedizin Rostock, Doberaner Str.142, 18057 Rostock, Germany; 8 Lukas Krankenhaus, Hindenburgstraße 56, 32257 Bünde, Germany; 9 CHU Amiens-Picardie, 1 Rond Point du Professeur Christian Cabrol, 80054, CEDEX 1 Amiens, France

**Keywords:** Total knee arthroplasty, UHMWPE, Gliding surface material, Clinical study

## Abstract

**Background:**

The gliding surface of total knee endoprostheses is exposed to high loads due to patient weight and activity. These implant components are typically manufactured from ultra-high molecular weight polyethylene (UHMWPE). Crosslinking of UHMWPE by ionizing radiation results in higher wear resistance but induces the formation of free radicals which impair mechanical properties after contact with oxygen. Medium-crosslinked UHMWPE enriched with vitamin E (MXE) provides a balance between the parameters for a sustainable gliding surface, i.e., mechanical strength, wear resistance, particle size, and oxidation stability. Therefore, a gliding surface for knee endoprostheses made up from this material was developed, certified, and launched. The aim of this study is to compare this new gliding surface to the established predecessor in a non-inferiority design.

**Methods:**

This multicenter, binational randomized controlled trial will enroll patients with knee osteoarthritis eligible for knee arthroplasty with the index device. Patients will be treated with a knee endoprosthesis with either MXE or a standard gliding surface. Patients will be blinded regarding their treatment. After implantation of the devices, patients will be followed up for 10 years. Besides clinical and patient-related outcomes, radiological data will be collected. In case of revision, the gliding surface will be analyzed biomechanically and regarding the oxidative profile.

**Discussion:**

The comparison between MXE and the standard gliding surface in this study will provide clinical data to confirm preceding biomechanical results in vivo. It is assumed that material-related differences will be identified, i.e., that the new material will be less sensitive to wear and creep. This may become obvious in biomechanical analyses of retrieved implants from revised patients and in radiologic analyses.

**Trial registration:**

ClinicalTrials.gov, NCT04618016. Registered 27 October 2020, https://clinicaltrials.gov/study/NCT04618016?term=vikep&checkSpell=false&rank=1.

All items from the World Health Organization Trial Registration Data Set can be found in Additional file 1.

**Supplementary Information:**

The online version contains supplementary material available at 10.1186/s13063-023-07811-1.

## Background

Total knee arthroplasty (TKA) is the only effective treatment for end-stage symptomatic knee damage caused by osteoarthritis or rheumatic arthritis. There is a trend towards younger and more active patients who receive a TKA, making higher demands towards their implant. Furthermore, a considerable share of (younger) TKA patients is overweight or obese [1, 2]. Higher activity levels and higher patient weight induce higher loads on the prosthesis components, especially on the meniscal component, which serves as gliding surface for articulation of the femoral component. Increased loads on the gliding surface may result in wear of the device material, leading to debris and aseptic loosening [3, 4]. This emphasizes the importance of the material properties on the outcome of TKA. After its introduction in the early 1960s, ultra-high molecular-weight polyethylene (UHMWPE) has represented the material of choice for the gliding surface [3]. Research on UHMWPE aimed to improve material properties and wear resistance. Improvements regarding wear resistance were achieved by crosslinking of UHMWPE through ionizing radiation. However, this process induces formation of free radicals in the material, which after contact with oxygen leads to oxidative degeneration, material embrittlement, and impairment of mechanical properties [5]. Remelting and annealing were introduced as post-irradiation treatments to improve oxidative stability of the material, but negative effects were induced by these treatments regarding mechanical properties [6]. Therefore, a method to prevent oxidation of crosslinked UHMWPE without modifying mechanical properties was searched for.

Vitamin E is an effective natural antioxidant, occurring in cell membranes and preventing phospholipids from being oxidized by free radicals [7]. Similarly, vitamin E prevents UHMWPE from irradiation-induced oxidation [8] and therefore represents a promising stabilizer of crosslinked UHMWPE. Two methods are deployed for supplementation of UHMWPE with vitamin E: blending, where the raw material is mixed with vitamin E powder before the irradiation and cross-linking process; and doping, where the irradiated implant is doped with vitamin E. Biomechanical data indicated that vitamin E-enriched crosslinked UHMWPE has an advantageous oxidation profile (also after aging) while maintaining mechanical properties important for the gliding surface of a TKA [9, 10, 8, 11].

In contrast to the application of highly crosslinked vitamin E-enriched UHMWPE for the liners in hip arthroplasty, where a large amount of clinical data is available [12–17], such data for TKA is rather rare. Flament et al. were the first to publish clinical results on vitamin E polyethylene bearings in TKA, providing good results for the device [18]. Takemura et al. compared clinical results of gliding surfaces made from highly crosslinked UHMWPE with and without vitamin E 2 years postoperatively, detecting no significant differences between both groups regarding alignment, Knee Society Score (KSS), complications, and radiolucent lines [19]. Likewise, Ftaita et al. compared clinical results of gliding surfaces made from conventional UHMWPE and highly crosslinked vitamin E-enriched UHMWPE, without any significant differences for complications, radiological results, forgotten joint score, and knee injury and osteoarthritis outcome score [20]. Using the same implants as in the studies discussed before, Orita et al. showed that highly crosslinked vitamin E-enriched UHMWPE gliding surfaces produced more, smaller and rounder wear particles compared to conventional UHMWPE, isolated from TKA patients’ synovial fluid [21]. Spece et al. performed a retrieval analysis of gliding surfaces made from highly crosslinked UHMWPE with and without vitamin E after rather short implantation times (mean 1.2–1.5 years) [22]. In the former group, the most prevalent reason for revision was instability (28.2%), followed by infection (23.3%) and aseptic loosening (17.5%), whereas in the group without vitamin E, infection (32.8%) was the most prevalent reason for revision, followed by aseptic loosening (25.4%) and instability (19.4%). Oxidation index was significantly increased in some areas of the retrievals without vitamin E and so were the articulating surface damage scores for burnishing, scratching, and pitting [22].

After extensive research in material science and biotribology a medium crosslinked, vitamin E-enriched gliding surface (MXE) was developed and launched in 2020. This material provides a balance between the parameters for a sustainable gliding surface, i.e., mechanical strength, wear resistance, particle size, and oxidation stability.

The aim of this study is the comparison of clinical outcome, oxidation profile, and wear analysis of MXE and the predecessor, ß-sterilized UHMWPE (ß-PE) for TKA. In this randomized controlled single blind clinical trial, it shall be shown that the new material is at least as efficient as the predecessor (non-inferiority).

## Methods/design

### Eligibility criteria and recruitment

Identification of eligible participants is performed by the investigators (surgeons) of the participating study centers from the general patients visiting their clinic for treatment of the indications mentioned. All patients designated for TKA with an e.motion UC Pro or e.motion PS Pro endoprosthesis (criteria see below) at the participating study centers will be screened for inclusion. The study proposal, including nature, purpose, and risks of the study, will be explained to the patient by the investigator during the preliminary consultation. A patient information sheet in layperson-comprehensible language is provided to the patient. The patient will be given sufficient time to consider the implications of the study before deciding whether to participate. Patients participating in the study must understand and agree with the patient information and be willing and able to participate in the study, including long-term follow-up visits. The written consent form will be obtained by one of the investigators of the study center.

There are no specific strategies for the identification of participants like advertisements or similar activities. Also, no coordinators are employed to identify eligible patients.

Eight centers in Germany and France are participating in the study, thereof are 3 academic hospitals (a list of the participating study centers is provided in Additional file 1). Study centers were accepted for study participation only after a successful site selection visit by the sponsor, where knowledge of and experience with the study devices was proven and ability to perform the study according to GCP principles was demonstrated.

The risk of not reaching the recruitment target is minimized by the inclusion of study centers which confirmed to be able to enroll 80 study patients during the recruitment phase of 18 months. By including several study centers, the risk of not reaching the recruitment target is minimized as the study protocol allows enrollment of up to 120 patients per study center in case that another study center will not reach the scheduled number of patients to be enrolled. To avoid center effects, the maximum number of patients to be enrolled by one study center is 120.

According to the instructions for use of the manufacturer, indications for an e.motion UC Pro and e.motion PS Pro endoprosthesis are as follows:

Severe knee joint disorders that cannot be treated through other therapies.

- Degenerative gonarthrosis
- Rheumatoid arthritis
- Posttraumatic arthrosis
- Symptomatic knee instability
- Knee stiffness
- Knee joint deformity

According to the instructions for use of the manufacturer, e.motion UC Pro and e.motion PS Pro endoprosthesis is contraindicated:

- In patients for whom reconstructive surgery to treat the joint disorder is an option
- In case of acute or chronic infections near the joint (systemic infections)
- In case of secondary diseases influencing the function of the joint implant
- In case of severe osteoporosis or osteomalacia with cementless implants
- In case of bone tumors in the region of implant fixation
- In case of poor bone quality and osseous malformations, diseases in the area of the implant fixation, which may primarily or subsequently affect the stability of the joint replacement anchorage
- In case of known hypersensitivity to the implant materials
- In all of the areas of application not listed under Indications

According to the study protocol, further exclusion criteria for the study are as follows:

- Age below 18 years or over 80 years
- American Society of Anesthesiologists (ASA) class ≥ 3
- Pregnancy

### Sample size

The aim of the present study is to show the non-inferiority of the MXE bearing material compared to the β-PE in terms of clinical and functional outcome, which is measured by the KSS. Lizaur-Utrilla et al. showed in their study that the minimally clinically important difference is at least 9 points for the KSS knee (kKSS) and at least 10 points for the KSS function (fKSS) [23]. In order to show non-inferiority of the new material, 10 points were selected as non-inferiority margin for sample size calculation. A recent publication showed that KSS results 5 years after primary TKA vary with a standard deviation of 30 points [24]. Thus, 30 points was used as standard deviation for sample size calculation.

Therefore, a two-sample *t* test with *α* = 0.05 and 80% power and a non-inferiority design (MXE vs. β-PE) with a non-inferiority margin of 10 points requires 224 patients in total (112 patients per group). Assuming a drop-out rate of 20% due to the long-term follow-up, in total 280 patients are necessary to meet the power criteria for the primary endpoint.

As the study includes two variations of the investigational device (e.motion UC Pro and e.motion PS Pro) together with the different bearing materials, in total, 560 patients will be randomized into this study. To avoid center effects, a maximum of 120 patients may be recruited per center. During study preparation, only centers were considered, which are able to recruit a sufficient number of patients according to their throughput of patients.

Patients who withdraw their consent for study participation will not be replaced by new patients. Intraoperative drop-outs due to deviation from the randomization protocol will be replaced by new patients.

### Randomization and blinding

For each participating center a separate randomization list be will prepared by the sponsor to avoid center specific effects and to assure a balanced (1:1) distribution of both treatments within one center (stratification). Randomization lists will be computer-generated using random block lengths.

**Fig. 1:**
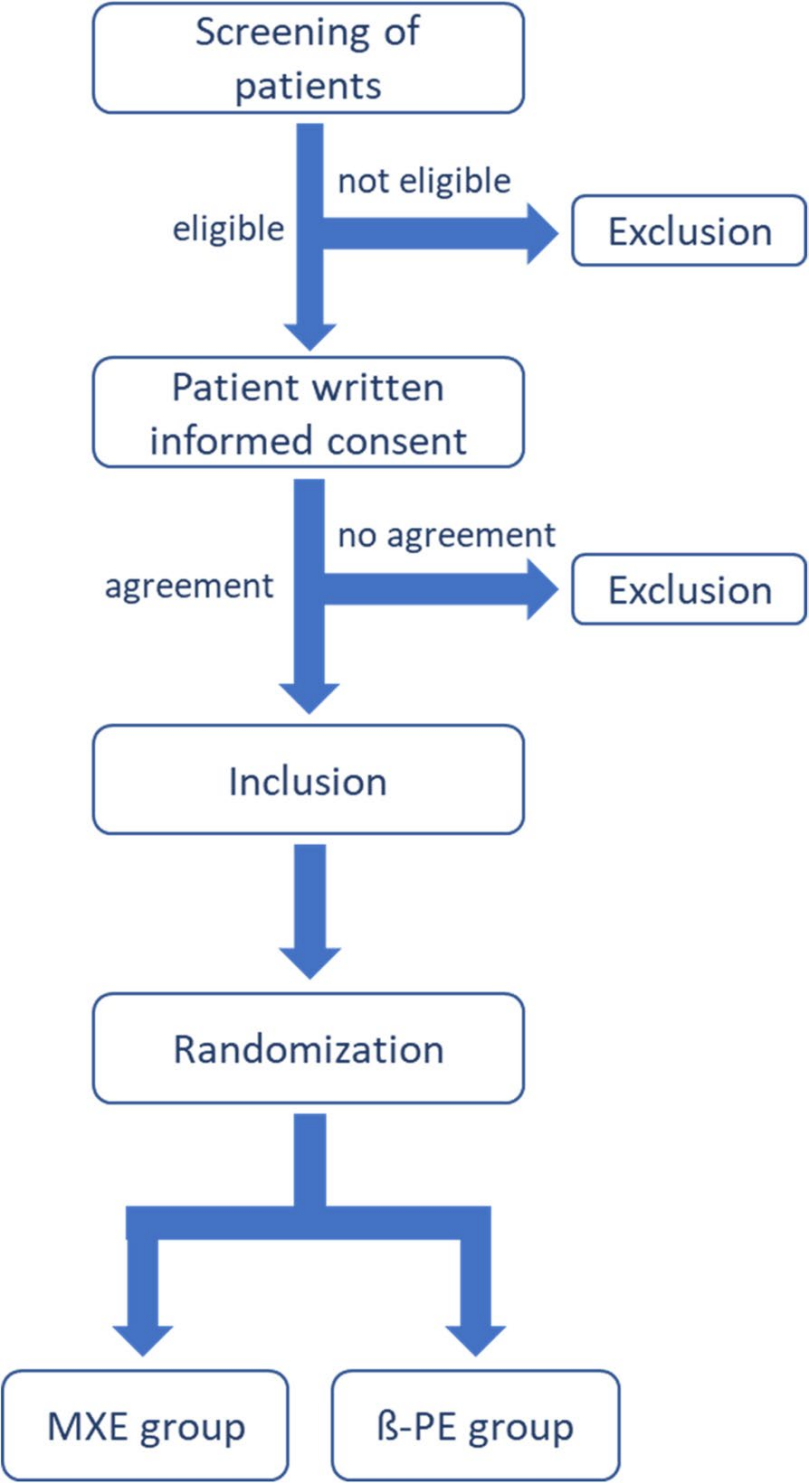
Patient inclusion flowchart

Randomization is taking place after the patient consented to be part of the clinical investigation (see Fig. 1). Intraoperatively, the sealed opaque envelope containing the information to use either the MXE or the β-PE gliding surface will be opened to randomly allocate the procedure for the specific type of bearing material and to ensure allocation concealment. Whether the study site is using the e.motion PS Pro or the e.motion UC Pro implant is independent from the randomization and according to the standard treatment of each site.

While study patients will be blinded, blinding of the surgeon is not possible due to the different color of the implants. Due to the infrastructure and capacity reasons in (some of) the participating study centers, the KSS is recorded by the investigators/surgeons, who typically performed the implantation of the devices. Therefore, blinding of outcome assessors cannot be ensured. The analyzation of radiological results will be performed in a blinded manner.

### Intervention

The implants are CE certified, and the TKA is performed according to the instructions for use. It will be further documented if patella resurfacing is performed, if the image-free navigation system OrthoPilot (Aesculap AG, Tuttlingen) is used, and if implants with or without the advanced surface technology (AS) are used.

In case of an intraoperative finding that the endoprosthesis is not indicated for a specific patient, the patients will be excluded from the study. Surgeries will be performed or, in cases of training procedures, supervised by board certified orthopedic surgeons. All centers meet the certification standards such as the German EndoCert system. Preoperative measures include digital planning of the prosthesis and standardized imaging, antiseptic, and anticoagulation standards. Furthermore, surgical approaches, anesthesia, and tourniquet use are performed according to the standards of each center. Postoperative regimen is not stipulated by the study plan to allow the study centers to follow their standard treatment regimen. It is expected that there are no major differences between the study centers regarding postoperative regimen. There may be minor differences between study centers, but as each study center includes patients in both study groups, the potential for cointervention bias is expected to be small.

Postoperative treatments including standardized weight bearing, physical therapy, anti-thrombotic medication, pain management, and discharge to rehabilitation centers accord to the standard proceeding in each study center. No difference is made between patients of the study groups or other patients not participating in the study.

No restrictions to any concomitant therapy or intervention are defined in this study. No additional surgical or interventional procedure is related to the study.

### Devices

The investigational device is the e.motion UC Pro or PS Pro knee endoprosthesis. The e.motion Pro system is indicated for patients requiring primary surgery. The e.motion UC Pro system is an ultracongruent posterior cruciate ligament retaining design. The e.motion PS Pro is a posterior stabilized posterior cruciate ligament sacrificing design. The size and type of the gliding surface (e.motion UC Pro or e.motion PS Pro meniscus component) depends on the femur component. The tibia has a safety stop which allows ± 30° of rotation. Both variations of the system are used within the study according to the preference and standard of care of the respective investigator and investigational site. Femur and tibia components are made up of a cobalt-chromium-molybdenum alloy. Femur and tibia components are available as AS variants, with a ceramic coating, which reduces wear and release of metal ions into the adjacent tissue and thereby may prevent allergic or hypersensitivity reactions [25–28].

The comparison in this study concerns the material of the gliding surface, either made from UHMWPE blended with 0.1% vitamin E, which is moderately crosslinked with < 50 kGy by γ-irradiation (MXE), or made from ß-sterilized UHMWPE (ß-PE).

### Outcomes

The primary goal of TKA is the restoration of the joint function and the reduction of pain. Measures to analyze these factors are included as outcomes in this study, to be able to compare the investigational device against the comparator.

#### Primary endpoint and measures

The primary endpoint of the study is the functional outcome of the patients 10 years postoperatively. The hypothesis of the study is that the new bearing material MXE is comparable to the established material in terms of performance and safety. Function of the knee after 10 years will be assessed using the KSS. The KSS is an established, examiner-administrated standard clinical evaluation tool reporting results for patients undergoing TKA. It separates findings in the operated knee (kKSS) score from findings related to the patient’s function (fKSS), which might be affected by co-morbidities. The two sub-scores are reported separately ranging from 0 points (worst result) to 100 points (best results), as well as summarized score, total KSS [29]. Final grading of total KSS results scores of 160 to 200 will be rated as excellent, 140 to 159 as good, 120 to 139 as fair, and less than 120 as poor.

#### Secondary endpoints and measures

The following secondary endpoints will be measured to evaluate the safety and performance of the investigational devices.

Preoperatively, data regarding demographics and anamnesis such as age, gender, weight, height, primary diagnosis, concurrent conditions, other joint restrictions, and ASA status will be collected and documented. Intraoperative and surgery-related data (e.g., duration of surgery, anesthesia, blood loss, length of hospital stay, used implant components incl. coating, and intraoperative complications) will also be collected and documented.

Survival of the implant (components) will be analyzed by Kaplan–Meier Analysis [30]. Survival in the sense of this study is defined as removal or exchange of any primary implant component including the removal or exchange of the meniscus component. Secondary interventions at the index knee, e.g., secondary patella replacement, do not count as a revision if no further exchange of the former implant components occur.

Clinical outcome will also be measured with the Oxford Knee Score (OKS), a 12-item, patient-reported questionnaire originally developed and validated specifically to assess function and pain in patients undergoing TKA. It is short, reproducible, valid, and sensitive to clinically important changes over time. OKS ranges from 0 to 48 with 48 being the best outcome [31].

Quality of life of the patients will be analyzed using the 5-dimension 5-level measure of the EuroQol Group (EQ-5D-5L), which is a simple and generic measure for clinical and economic assessment. The questionnaire covers five different dimensions (mobility, self-care, usual activities, pain/discomfort, anxiety/depression) rated in 5 levels (from “the worst health you can imagine” to “the best health you can imagine”) [32]. The questionnaire is filled by the patients themselves.

Radiographic evaluation will be used to evaluate the implant status as well as device condition and potential presence of device-related adverse events including fracture, wear, loosening, or radiolucencies. An external x-ray laboratory will perform the analysis and evaluation of radiographs. In particular, radiolucencies at the interface between the implant and bone, migration of the femur and tibia component in relation to the bone, femoral-tibial angle, mechanical lateral femoral angle, and mechanical medial tibial angle will be analyzed. Furthermore, a new method combining 2-dimensional–3-dimensional registration and artificial intelligence will be applied, which enables accurate determination of changes in the postoperative polyethylene wear in situ. The precision and accuracy of this method is comparable to radiostereometric analysis [33].

Oxidation profile and wear analysis of available retrievals will be analyzed by the biomechanical laboratory of the sponsor. Special focus of these analyses is the oxidation profile, the wear behavior, the mechanics, and the cumulative linear abrasion of these retrievals. These measurements will provide in vivo data regarding the implant material in addition to detailed in vitro data already available [34, 35, 11].

During the course of the study, any upcoming intraoperative or postoperative (serious) adverse device events or effects related or not related to the product under investigation will be documented. The total number of adverse events will be summarized and further evaluated by the sponsor and reported according to local legislation and necessity. Recorded complications will be categorized and analyzed to assess the safety of the investigational product.

In case a patient requires revision of the primary implanted knee endoprosthesis, additional information on the revision surgery will be collected. The reason for revision together with the clinical symptoms shall be documented. Further examinations (e.g., arthroscopy) prior to the revision and the date of revision surgery will be documented. If pre-revision radiographs are taken as part of the clinical routine, the results of the radiographic analysis will be documented.

### Data collection, management, statistical analysis and monitoring

The data will be collected by the investigators from routine examinations and documented in an electronic database system. Data entered in the electronic database is pseudonymized using a unique patient identification number to keep the trial patients’ confidentiality. Table 1 shows which data is acquired during the course of the study. In case of death or discontinuation of a patient, it will be inquired whether the knee endoprosthesis was in situ at that time (survival analysis). Trial management, including monitoring of data and data quality, is performed by the sponsor.

**Table 1:**
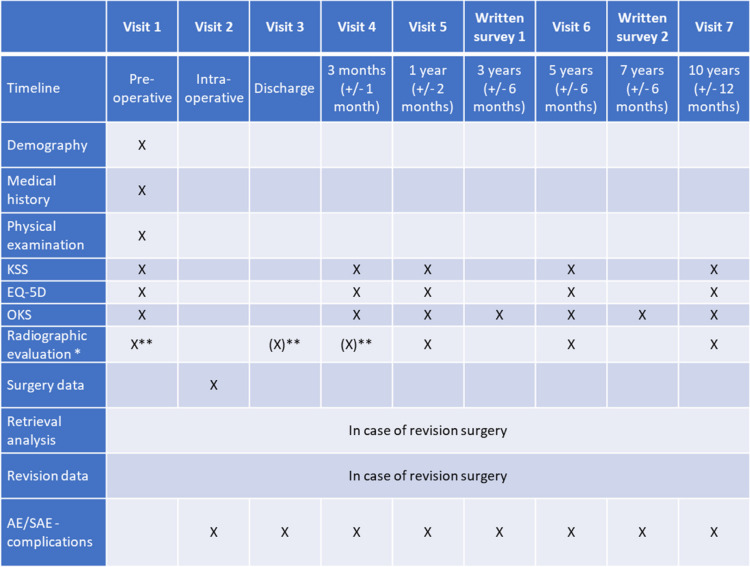
Data acquisition during the course of the study

^*^Required images: knee AP (standing), knee lateral (standing)

^**^Additionally: long leg (standing)

(X): radiographic evaluation may be performed at either visit

There are no specific plans to promote participant retention and complete follow-up. The patients are informed about the follow-up visits before their decision to participate in the study. The visits are kept to a minimum (3 months, 12 months, 5 years, 10 years) and accord to typical follow-up examinations also performed in non-study patients. In addition, patients will receive the OKS self-assessment score at 3 and 7 years postoperatively, to reduce loss-to follow-up rate and allow better monitoring of study patients. Patients will be requested to send the OKS questionnaire back to the study center.

Reasonable effort should be made by the study centers to contact any patient lost to follow-up during the course of the study in order to complete assessments and retrieve any outstanding data and study supplies.

The final programming will be performed after closure of the database by use of an appropriate statistical software package SAS. The sponsor has an overview of results from all participating centers. All data will be analyzed by means of appropriate tables, figures, listings, and statistical tests. Missing data will be analyzed as such and will not be replaced by estimates. The statistical analysis of the primary endpoint has a confirmative character, whereas the secondary endpoints will be analyzed explorative. This must be considered when interpreting the *p*-values and confidence intervals. The description of the patient cohort in means of demographic data and the baseline values of investigated parameters will take place as whole as well as grouped by the therapy group. Analyses will be performed according to the intent-to-treat principle. The consistency of the results will be tested by per-protocol analysis (sensitivity analysis).

The study hypothesis will be tested using a two-sample *t* test. The results of the KSS Score will be analyzed as sum of the sub scores fKSS and kKSS. Primary testing will be done comparing the MXE with the β-PE group. Comparison between the different implant types might be done exploratively. Each implant variation (UC Pro vs. PS Pro) will be tested individually for primary endpoint analysis. Secondary endpoints will be tabulated as frequencies and rates/results respectively as means and standard deviations. Confidence intervals will be used when appropriate.

An explorative interim analysis of secondary objectives will be done 1 and 5 years postoperatively. It is intended to publish the 1-year as well as the 5- and 10-year results in a peer-reviewed journal in order to contribute to publicly available knowledge. Furthermore, the study and (intermediate) results may be presented on scientific conferences. Therefore, data of all available patients and centers shall be included. Manuscripts shall be reviewed and approved by all participating study centers. In case that the study results cannot be published in a peer-reviewed journal (e.g., due to non-significant results), a manuscript shall be deposited in a preprint archive. Furthermore, a study report will be attached to the ClinicalTrials.gov entry of this study.

Authorized, qualified representatives of the sponsor or designated personnel of a contract research organization will perform monitoring visits at investigational sites in regular intervals to verify adherence to protocol and local legal requirements, to perform source data verification and to assist the investigator in study related activities. Approximately one visit per site and year is planned, with more visits in the beginning of the study. As a risk-based approach, discovered deviations from the study protocol may increase the number or intensity of monitoring visits. Site audits are not generally planned. If monitoring activities reveal the necessity, e.g., due to severe deviations from GCP principles, site audits may be performed.

Vendors employed for the study were audited and approved by the processes of the sponsor supplier quality management.

## Discussion

The advancement of UHMWPE as a bearing material in the past 60 years can be considered as an evolution, adjusting the characteristics of the material to the function of the devices. Wear resistance, mechanical strength, oxidative stability, and the size of released particles are critical parameters influencing the safety and performance of the devices. MXE reveals a beneficial balance between these parameters and showed improved biomechanical properties in vitro in comparison to other established bearing materials. The comparison between MXE and ß-PE in this study will provide clinical data to confirm the biomechanical results in vivo.

It is assumed that material-related differences will be obtained, i.e., that the new material will be less sensitive to wear and creep. This may become obvious in radiologic analyses, where a new approach will be employed to measure wear of prosthesis components in situ [36, 33] and in biomechanical analyses of retrieved implants from revised patients. Whether these differences will lead to better clinical and functional outcomes or less complications and higher survival is in question and will be investigated thoroughly.

This study protocol features the history of development in knee arthroplasty technology from basic material sciences through biotribology and a final device to certification and application in humans. Through this study, data required by biomechanical testing can be confirmed or refuted, depending on whether there will be an advantage of the new material or not. This again may add knowledge to development and testing methods.

An apparent strength of this study is the comparison of two devices which only differ in their material but not in shape, dimensions, application, or other aspects. In combination with the new approach for the radiologic analysis to measure wear of prosthesis components in situ, the study performed according to this protocol is expected to deliver relevant results in the field of knee arthroplasty and implant technology.

## Trial status

This manuscript corresponds to version 1 of the approved study protocol. The study started in February 2021 with first patient in on 8 March 2021. To date (May 2023), 435 patients are included in the study. Last patient in is expected in December 2023, and, therefore, last patient out is expected in Q4 2033. Interim analyses will be performed as discussed above.

### Supplementary Information

**Additional file 1. **WHO Trial Registration Data Set (Version 1.3.1) and study center information.

**Additional file 2. **Patient information.

## Data Availability

Any data required to support the protocol can be supplied on request.

## Abbreviations

AS: Advanced surface
ASA: American Society of Anesthesiologists
ß-PE: ß-sterilized UHMWPE
EQ-5D-5L: 5-Dimensions 5-level measure of the EuroQol Group
fKSS: Knee Society Score function
kKSS: Knee Society Score knee
KSS: Knee Society Score
MXE: Medium crosslinked vitamin E-enriched UHMWPE
OKS: Oxford Knee Score
TKA: Total knee arthroplasty
UHMWPE: Ultra-high molecular weight polyethylene
