# Supplementary material for: Vitamin E-enriched medium cross-linked polyethylene in total knee arthroplasty (VIKEP): clinical outcome, oxidation profile, and wear analysis in comparison to standard polyethylene—study protocol for a randomized controlled trial
Source: Trials. 2024 Jan 5;25:27. doi: 10.1186/s13063-023-07811-1 (PMC10768156; doi:10.1186/s13063-023-07811-1)
Supplement: Supplementary file 1 — Additional file 1. WHO Trial Registration Data Set (Version 1.3.1) and study center information. [file 13063_2023_7811_MOESM1_ESM.docx]

Appendix 1

Vitamin E enriched medium cross-linked polyethylene in total knee arthroplasty (VIKEP): clinical outcome, oxidation profile and wear analysis in comparison to standard polyethylene: study protocol for a randomized controlled trial

## WHO Trial Registration Data Set (Version 1.3.1)

Table 1: All items of the WHO Trial Registration Data Set (Version 1.3.1)

| Primary Registry and Trial Identifying Number | ClinicalTrials.gov NCT04618016 |
| --- | --- |
| Date of Registration in Primary Registry | 2020-11-05 |
| Secondary Identifying Numbers | AAG-O-H-1906 |
| Source(s) of Monetary or Material Support | Aesculap AG |
| Primary Sponsor | Aesculap AG |
| Secondary Sponsor(s) | NA |
| Contact for Public Queries | MS |
| Contact for Scientific Queries | WM |
| Public Title | Evaluation of Medium Cross-linked Polyethylene With and Without Vitamin E for Total Knee Arthroplasty (VIKEP) |
| Scientific Title | Prospective, Randomized, Single-blind, Multinational, Long-term Study for the Evaluation of the Clinical Outcome, Oxidation Profile and Wear Analysis of Medium Cross-linked Polyethylene With and Without Vitamin E for Total Knee Arthroplasty |
| Countries of Recruitment | Germany |
|  | France |
| Health Condition(s) or Problem(s) Studied | Osteoarthritis, Knee |
|  | Arthritis, Rheumatoid |
|  | Intra-Articular Fractures |
|  | Joint Instability |
|  | Genu Valgum or Varum |
| Intervention(s) | Device: UHMWPE with Vitamin E; e.motion Pro (PS and UC) with MXE gliding surface |
|  | Device: UHMWPE without Vitamin E; e.motion Pro (PS and UC) with β-PE gliding surface |
| Key Inclusion and Exclusion Criteria | Ages eligible for study: 18 Years to 80 Years (Adult, Older Adult ); Sexes eligible for study: all; Accepts healthy volunteers: no; Sampling method: Probability Sample |
|  | Inclusion criteria: Indication for a total knee replacement with a mobile-bearing UC or PS inlay; Written informed consent for participating at the clinical study; Willingness and mental ability to participate at the long-term follow-up examinations |
|  | Exclusion criteria: Patient age < 18 and > 80 years; Pregnancy; High risk patients (ASA class >3) |
| Study Type | Observational |
|  | Allocation: randomized |
|  | Masking: single-blind |
|  | Assignment: parallel |
|  | Purpose: Therapy |
| Date of First Enrollment | 2021-03-08 |
| Sample Size | 560 [Anticipated] |
| Recruitment Status | Recruiting |
| Primary Outcome(s) | Clinical Outcome measured with the Knee Society Score 10 years postoperatively |
| Key Secondary Outcomes | Survival rate until the last follow-up at 10 years postoperatively |
|  | Progress of Clinical Outcome (Oxford Knee Score) compared to baseline; preoperatively, 3 months, 12 months, 3 years, 5 years, 7 years, 10 years |
|  | Quality of Life (EQ-5D-5L); preoperatively, 3 months, 12 months, 5 years, 10 years |
|  | Comparison of Radiographic evaluation over time; preoperatively, directly postoperatively, 3 months, 12 months, 5 years, 10 years |
|  | Oxidation profile and wear analysis of available retrievals; up to 10 years postoperatively |
| Ethics Review | Primary positve vote: Ethikkommission an der Universitätsmedizin Rostock (A 2020-0171); 2020-07-28 |
|  | Positively rated by the corresponding ethics committee of each participating center |
| Completion date | 2033-12 (Estimated) |
| Summary Results | NA |
| IPD sharing statement | NA |

## Study centers

Table 2: Study centers and corresponding ethics committees

| Site | Center | Ethics Committee |
| --- | --- | --- |
| 01 | Orthopädische Klinik und Poliklinik  Universitätsmedizin Rostock  Doberaner Straße 142  18057 Rostock | Ethikkommission an der Universitätsmedizin Rostock;  A 2020-0171 |
| 02 | Waldkliniken Eisenberg  Deutsches Zentrum für Orthopädie  Klosterlausnitzer Straße 81  07607 Eisenberg | Ethik-Kommission Universitätsklinikum Jena;  2020-1882-BO Zweitvotum |
| 03 | Krankenhaus Reinbek St. Adolf-Stift  Orthopädie und Unfallchirurgie  Hamburger Straße 41  21465 Reinbek | Ethikkommission bei der Ärztekammer Schleswig-Holstein;  111/20m |
| 04 | Not selected for study participation | NA |
| 05 | Park-Klinik Weißensee  Orthopädie und Unfallchirurgie  Schönstraße 80  13086 Berlin | Ethik-Kommission Ärztekammer Berlin;  Eth-MZ 18/20 |
| 06 | CHU Amiens-Picardie  Chirurgie Orthopédique et traumatologique  Site Sud – Entrée Principale  1 Rond-Point du Professeur Christian Cabrol  80054 Amiens cedex 1, France | Comité de Protection des Personnes Nord Ouest III;  Réf. CPP 2021-31  No IDRCB : 2020-A03411-38 |
| 07 | CHU de Grenoble  Chirurgie de l'arthrose et du sport, urgences traumatiques des membres  Avenue Maquis du Grésivaudan,  38700 La Tronche, France | Comité de Protection des Personnes Nord Ouest III;  Réf. CPP 2021-31  No IDRCB : 2020-A03411-38 |
| 08 | Lukas-Krankenhaus Bünde Unfallchirurgie und Orthopädie, Handchirurgie Hindenburgstraße 56 32257 Bünde | Ethik-Kommission der Ärztekammer Westfalen-Lippe und der Westfälischen Wilhelms-Universität;  2021-749-b-S |
| 09 | Brüderhaus Koblenz Kliniken für Orthopädie & Unfallchirurgie Kardinal-Krementz-Straße 1-5 56073 Koblenz | Ethik-Kommission Landesärztekammer Rheinland-Pfalz; 2022-16502 |
