# Supplementary material for: Vitamin E-enriched medium cross-linked polyethylene in total knee arthroplasty (VIKEP): clinical outcome, oxidation profile, and wear analysis in comparison to standard polyethylene—study protocol for a randomized controlled trial
Source: Trials. 2024 Jan 5;25:27. doi: 10.1186/s13063-023-07811-1 (PMC10768156; doi:10.1186/s13063-023-07811-1)
Supplement: Supplementary file 2 — Additional file 2. Patient information. [file 13063_2023_7811_MOESM2_ESM.docx]

Appendix 2

# Vitamin E enriched medium cross-linked polyethylene in total knee arthroplasty (VIKEP): clinical outcome, oxidation profile and wear analysis in comparison to standard polyethylene: study protocol for a randomized controlled trial

## Example patient information (German)

**P A T I E N T E N I N F O R M A T I O N**

**Prospektive, randomisierte, einfach verblindete Langzeitevaluation zum klinischen Ergebnis, dem Oxidationsprofil und der Abriebsanalyse bei moderat vernetztem Polyethylen mit oder ohne Vitamin E Zusatz beim künstlichen Kniegelenksersatz**

*Originaltitel: Prospective, randomized, single-blind, multinational, long-term study for the evaluation of the clinical outcome, oxidation profile and wear analysis of medium cross-linked Polyethylene with and without Vitamin E for total knee arthroplasty*

**Studienabkürzung: VIKEP**

Sehr geehrte/r Patient/in,

anhand Ihrer Krankengeschichte wurde festgestellt, dass Sie zur Teilnahme an der oben genannten Studie in Frage kommen. Wir möchten Sie daher bitten, die folgende Information zu lesen und zu entscheiden, ob Sie damit einverstanden sind, Ihre Daten für diese wissenschaftliche Erhebung zur Verfügung zu stellen.

In dieser Studie möchten wir Informationen zu dem Kniegelenk e.motion Pro und der dazugehörigen Polyethylen Gleitfläche mit und ohne Vitamin E Zusatz erheben und evaluieren, wie gut Sie als Patient/-in hiermit im Alltag zurechtkommen und welche Unterschiede sich bei der Standardbehandlung von Patienten im zeitlichen Verlauf zwischen den beiden Materialvarianten zeigen.

Die Verwendung von Informationen über Ihre Gesundheit unterliegt gesetzlichen Bestimmungen und setzt voraus, dass Sie transparent und vollständig durch Ihren Studienarzt über die vorliegende Studie informiert werden. Um Ihre persönlichen Daten weitergeben und wissenschaftlich nutzen zu können, müssen Sie in deren Nutzung freiwillig einwilligen. Sollten Sie die Teilnahme an dieser Studie ablehnen, entstehen Ihnen daraus keinerlei Nachteile.

Die Studie wird von der Aesculap AG (78532 Tuttlingen, Deutschland), dem Medizinproduktehersteller der Knieendoprothese e.motion Pro, als studienverantwortlichem Sponsor organisiert und finanziert.

**Was ist das Knieimplantat e.motion Pro?**

Die Kniegelenksersatzoperation gehört zu den häufigsten orthopädischen Eingriffen in Deutschland. Bei einer knieendoprothetischen Gelenkversorgung wird das erkrankte und schmerzhafte Kniegelenk durch ein künstliches Knieimplantat ersetzt. Dieses besteht in der Regel aus einer Ober- und Unterschenkelkomponente, sowie einer Gleitfläche, welche die Funktion der Menisken übernimmt. Diese Gleitfläche besteht aus Polyethylen, einem speziellen Kunststoff, der mit Hilfe von ionisierender Strahlung so stabilisiert wird, dass er speziell in der Endoprothetik angewendet werden kann. Ziel ist es, den Eingriff so schonend wie möglich für das umliegende Gewebe zu gestalten und so viel gesunden Knochen wie möglich zu erhalten. Die Prothese soll gut in den umliegenden Knochen integriert werden und eine Lastübertragung erreichen, die der natürlichen Lastübertragung im gesunden Kniegelenk möglichst nahe kommt.

In dieser Beobachtungsstudie sollen die Ergebnisse der e.motion Pro Prothese erfasst werden. Bei dem künstlichen Kniegelenk e.motion Pro handelt es sich um ein modernes Implantat, dass bereits einige Jahre erfolgreich international implantiert wird. Dabei kann Ihr behandelnder Arzt individuell entsprechend Ihrer anatomischen Voraussetzungen zwischen einer kreuzbanderhaltenden oder kreuzbandersetzenden Implantatvariante wählen.

Der Hersteller dieser Prothese, die Aesculap AG (Tuttlingen, Deutschland), hat für dieses Produkt das Material der Gleitfläche weiterentwickelt und bietet eine neue Generation der Gleitfläche der e.motion Pro Prothese an. Dabei wird dem Kunststoff Vitamin E als Stabilisator, ein sogenanntes Antioxidationsmittel, hinzugefügt, um die Abriebseigenschaften sowie die Langzeitstabilität zu fördern. Dabei soll das Vitamin E das Material im Körper vor freien Radikalen schützen und die ursprünglichen Materialeigenschaften so lange wie möglich aufrecht erhalten. Dieses Konzept findet bereits seit einigen Jahren erfolgreiche Anwendung in der Hüftendoprothetik und soll nun auf die Knieendoprothetik übertragen werden.

Dafür werden im Rahmen dieser Studie die beiden Varianten der Gleitfläche mit und ohne Vitamin E verglichen, um potenzielle Vorteile der Vitamin E Zugabe in der Routineanwendung zu beurteilen.

**Was ist das Ziel dieser Beobachtungsstudie?**

Eine Beobachtungsstudie dient dazu, Informationen über ein Medizinprodukt zu erfassen, dass in der klinischen Routine und entsprechend der gängigen Praxis eingesetzt wird. Die beiden Gleitflächen ß-PE (ohne Vitamin E) und MXE (mit Vitamin E) zusammen mit dem künstlichen Kniegelenk e.motion Pro sind bereits CE-zertifizierte und zugelassenes Medizinprodukte. Das bedeutet, dass Sie im Rahmen dieser Beobachtungsstudie keinen zusätzlich belastenden, studienbedingten medizinischen Untersuchungen oder Behandlungsmaßnahmen unterzogen werden. Ihre Behandlung erfolgt ausschließlich gemäß dem medizinischen Standard. Die Studie dient daher nur dazu, diese standardgerechte Behandlung zu beobachten und speziell wissenschaftlich zu dokumentieren.

Um ausreichend Daten für aussagekräftige Ergebnisse zu sammeln, werden an dieser Beobachtungsstudie bis zu 560 Patienten über einen Zeitraum von 1,5 Jahren in fünf Krankenhäusern in Deutschland und in zwei Krankenhäusern in Frankreich teilnehmen. Dabei werden in allen Kliniken beide Varianten der Gleitfläche zufällig verteilt angewendet. Um möglichst objektive, qualitativ hochwertige Ergebnisse zu erhalten, werden Sie als Patient bei der Teilnahme an der Studie zufällig („randomisiert“) zu einem dieser Behandlungsarme zugeteilt und erhalten entweder eine Gleitfläche mit oder ohne Vitamin E. Ihre eigentliche Behandlung unterscheidet sich dadurch nicht von der, die Patienten außerhalb der Studie bekommen. Alle sonstigen medizinischen Entscheidungen obliegen selbstverständlich Ihrem behandelnden Arzt und unterscheiden sich nicht von der Standardtherapie.

Die gesammelten Daten werden in pseudonymisierter, also verschlüsselter, Form (d.h. Ihre Daten werden einer Nummer zugeordnet ohne Ihren Namen, Initialen, Adresse oder Geburtsdatum zu notieren) auf einem Papierbogen gesammelt oder teilweise direkt in einer elektronischen Datenbank online abgelegt, um diese später wissenschaftlich auswerten zu können.

Wenn Sie an dieser Beobachtungsstudie teilnehmen möchten, wird Ihr Arzt die geplante Behandlung für die wissenschaftliche Analyse dokumentieren. Rechtsgrundlage für diese Dokumentation ist Ihre freiwillige Einwilligung.

**Was wird in dieser Studie genau gemacht?**

Es wird erfasst, wie der Zustand Ihres Kniegelenks vor der Operation war, der Verlauf der OP selbst und die Ergebnisse der anschließenden Untersuchungen nach 3 Monaten sowie 1, 5 und 10 Jahren. Es werden Basisdaten und medizinische Hintergründe zu Ihrer Person dokumentiert, die eine Einschätzung und Bewertung der Studienergebnisse erlauben. Die Untersuchungen werden in der Klinik stattfinden, in denen Sie Ihr künstliches Kniegelenk erhalten haben. Daher werden Sie von Ihrem Operateur nach diesen Zeitpunkten zu routinemäßigen Nachkontrollen eingeladen und Ihre Daten für eine wissenschaftliche Auswertung erfasst. Bei diesen klinischen Untersuchungen werden Sie einen Fragebogen erhalten, in dem Sie ausführlich zu Ihrer Zufriedenheit mit ihrem Kniegelenk im Alltag und zu Schmerzen befragt werden. Teile dieser Fragebögen werden Sie auch zwischen den angegebenen Nachuntersuchungszeitpunkten nach 3 und 7 Jahren per Brief erhalten. Außerdem wird Ihr zuständiger Arzt Sie ausführlich untersuchen und die Funktionalität und Beweglichkeit Ihres Gelenkes medizinisch bewerten. Die im klinischen Standard erstellten Röntgenbilder, sowie die körperlichen Untersuchungen werden von Ihrem behandelnden Arzt pseudonymisierten auf einem elektronischen Dokumentationsbogen festgehalten. Dies gilt für alle erhobenen Daten. Sollte es im zeitlichen Verlauf notwendig sein, dass Ihr Implantat gewechselt werden muss, wird der Operateur die entfernte Gleitfläche an den Hersteller schicken, um eine detaillierte biomechanische Analyse des Materials vorzunehmen. Auch hier werden Sie als Patient zu keinem Zeitpunkt persönlich identifizierbar sein.

Die vor- und nachbereitenden Behandlungen entsprechen der üblichen Routine im Rahmen der Implantation eines künstlichen Kniegelenks und unterscheiden sich bei der Teilnahme an dieser Studie nicht.

**Was bedeutet Randomisierung und Verblindung?**

Bei der Operation, wird per Zufall (Randomisierung) entschieden, welche Kunststoff Gleitfläche bei Ihnen verwendet wird. Dabei handelt es sich wie oben beschrieben um zwei für Patienten zugelassene Polyethylen Gleitflächen ohne und mit Vitamin E Zusatz. Ihr Studienarzt wird hierfür einen entsprechenden Randomisierungsumschlag öffnen, der Sie zufällig zu einer dieser beiden Gruppen zuteilt. Auf die Auswahl der sonstigen Implantatkomponenten wird kein Einfluss genommen.

Verblindung bedeutet, dass im Rahmen der Studie Ihnen als Studienteilnehmer nicht mitgeteilt wird, in welchem Behandlungsarm Sie sich befinden. Dies soll vor allem dazu führen, dass Sie möglichst unbeeinflusst die notwendigen Angaben zur Funktionalität und Lebensqualität mit Ihrem Kniegelenk beschreiben können. Ihr behandelnder Arzt weiß jedoch in welchem Behandlungsarm Sie sich befinden.

**Versicherungsschutz**

Die Behandlung selbst und die verwendeten Implantatkomponenten von e.motion Pro sowie die Behandlungsrisiken entsprechen der Standardversorgung. Für die e.motion Pro Knieprothese besteht eine gesetzliche Produkthaftpflichtversicherung des Herstellers.

Um eventuelle Wegeunfälle abzusichern, die einem Studienpatienten auf dem Weg zu der Klinik oder von der Klinik nach Hause im Zuge eine Nachuntersuchung passieren könnten, stellt der Studiensponsor (Aesculap AG) freiwillig eine Wegeversicherung für die an der Studie teilnehmenden Patienten bereit. Sollten Sie einen Unfall auf dem Weg von oder zu einer der Untersuchungen im Rahmen Ihrer Nachbeobachtung erleiden, nehmen Sie bitte unter folgenden Kontaktdaten direkt Kontakt mit dem Versicherer, gegebenenfalls mit Unterstützung durch Ihren Studienarzt, auf:

HDI Global SE

Niederlassung Hannover

Riehorststr. 4

30659 Hannover

Policen-Nr.: 35-005049-03459

Sofern Sie Ihre Anzeige direkt an den Versicherer richten, informieren Sie bitte zusätzlich Ihren Studienarzt. Die Versicherungsbedingungen werden Ihnen bei einer Studienteilnahme vom Studienarzt ausgehändigt.

**Was passiert mit Ihren Daten?**

Ihr Arzt wird Ihre für die Studie relevanten Gesundheits- und Behandlungsdaten pseudonymisiert auf einem Papierbogen oder in einer elektronischen Datenbank direkt digital erfassen und an den Sponsor der Studie, die Aesculap AG weiterleiten.

Ihre pseudonymisierten Daten werden vom Studienarzt oder besonders zur Verschwiegenheit verpflichteten Personen auf einem webbasierten elektronischen Datenerfassungssystem der Firma InterActive System (SecuTrial) gespeichert. Diese Daten werden später wissenschaftlich ausgewertet. Der Sponsor (Aesculap AG) der Studie hat den aktuellen Dienstleister mit der Bereitstellung der Datenbank beauftragt und in einer vertraglichen Auftragsverarbeitung nach Art. 28 Datenschutzgrundverordnung (DSGVO) verpflichtet. Der Sponsor (Aesculap AG) behält sich das Recht vor, den Dienstleister der elektronischen Datenbank im Verlauf der Studie zu wechseln.

Ihre Röntgenbilder wird Ihr behandelnder Arzt verschlüsselt an die Plattform „UNITY“ der Raylytic GmbH (Leipzig, Deutschland) übermitteln, damit diese dort gesammelt und zu einem späteren Zeitpunkt wissenschaftlich ausgewertet werden können. Die Raylytic GmbH ist ein Zentrallabor, welches zur Sicherstellung einer größtmöglichen Objektivität insbesondere radiologische Daten in pseudonymisierter Form analysiert.

Zugriff auf diese Daten zur ordnungsgemäßen Studiendurchführung bzw. -überwachung, haben nur zur Verschwiegenheit verpflichteten Personen (Monitore), die entweder dem Sponsor angehören oder die vom Sponsor mit der wissenschaftlichen Analyse der Daten beauftragt wurden. Außer Ihrem Studienarzt kann keine dieser Personen eine Verbindung zwischen den Daten und Ihrer Person herstellen. Alle an der elektronischen Dokumentation auf SecuTrial und UNITY beteiligten Personen sind streng zur Verschwiegenheit verpflichtet. Der Datenschutz und mithin die Einhaltung des geltenden Datenschutzrechts werden jederzeit gewährleistet. Ebenso können zuständige Behörden (wie z.B. BfArM etc.) Einsicht in Ihre verschlüsselten Gesundheitsdaten und Ihre Patientenakte bekommen.

In der Klinik ist der Zugang zu Ihren auf SecuTrial und UNITY gespeicherten Daten auf Ihren behandelnden Arzt und sein Personal beschränkt. Diese Personen erhalten einen Zugangscode für die Datenbank, welchen sie vertraulich behandeln müssen und nicht weiterreichen dürfen. Jeder Zugriff auf die Datenbank wird dokumentiert.

Die Datenübermittlung auf SecuTrial und UNITY erfolgt mit der gleichen Sicherung (SSL-gesicherte HTTPS Datenfernverbindung), die z.B. bei Kreditkartengeschäften im Internet ebenfalls verwendet wird.

Für die auf SecuTrial und UNITY erhobenen Daten ist die Aesculap AG verantwortlich. Sollten Unsicherheiten zu den erhobenen Daten bestehen oder Sie noch weitere Fragen diesbezüglich haben, wenden Sie sich bitte an Ihren Studienarzt. Sie können jederzeit Ihre Einwilligung zu der Teilnahme an der Studie und/oder zur Erhebung und Verarbeitung Ihrer personenbezogenen Daten ohne Angabe von Gründen (mündlich oder schriftlich) widerrufen, ohne dass Ihnen dadurch Nachteile für Ihre medizinische Behandlung entstehen. Ihren Widerruf richten Sie bitte direkt an Ihren Studienarzt.

Es ist geplant, die Ergebnisse und Teilergebnisse der Studie zu veröffentlichen. Natürlich sind die veröffentlichten Daten anonymisiert, so dass die Rückverfolgung zu einzelnen Patienten nicht mehr möglich ist. Zum Zwecke der Überprüfung korrekter wissenschaftlicher Auswertungen kann es notwendig sein, Ihre pseudonymisierten Daten an eine Redaktion einer wissenschaftlichen Fachzeitschrift weiterzugeben.

**Welche Rechte haben Sie im Bezug auf die Verarbeitung Ihrer Daten?**

**Recht auf Auskunft**

Sie haben das Recht auf Auskunft über die Sie betreffenden Daten, die im Rahmen der Studie durch die Aesculap AG, verarbeitet werden (z. B. durch Aushändigen einer kostenfreien Kopie) (Art. 15 DSGVO).

**Recht auf Berichtigung**

Sie haben das Recht auf Berichtigung und/oder Vervollständigung, sofern die Sie betreffenden verarbeiteten Daten unrichtig oder unvollständig sind. Die Berichtigung wird sodann unverzüglich vorgenommen (Art. 16 DSGVO).

**Recht auf Löschung**

Sie haben unter den Voraussetzungen des Art. 17 DSGVO das Recht auf Löschung Sie betreffender Daten.

**Recht auf Einschränkung der Verarbeitung**

Sie haben unter den Voraussetzungen des Art. 18 DSGVO das Recht, die Einschränkung der Verarbeitung durch die Aesculap AG zu verlangen, d.h. die Daten dürfen nur gespeichert, nicht jedoch weiter verarbeitet werden.

**Recht auf Datenübertragbarkeit**

Sie haben das Recht, die sie betreffenden Daten in maschinenlesbarer Form zu erhalten. Sie können verlangen, dass diese Daten entweder Ihnen oder, soweit technisch möglich, einer anderen von Ihnen benannten Stelle übermittelt werden (Art. 20 DSGVO).

**Recht auf Widerspruch**

Wenn wir Ihre personenbezogenen Daten verarbeiten , weil wir Ihre Daten auf Basis eines berechtigten Interesse verarbeiten, haben Sie das Recht, der Verarbeitung zu widersprechen.

Ihre vorstehenden Rechte auf Auskunft, Berichtigung und Einschränkung der Verarbeitung und Widerspruch können aber insofern eingeschränkt sein, soweit es die Umsetzbarkeit der oben genannten Studie ernsthaft beeinträchtigen oder unmöglich machen würde. Sofern Sie von Ihrem Recht auf Löschung nicht Gebrauch machen, werden Ihre bis zum Widerruf erhobenen Daten weiterhin in der Auswertung verwendet, um die Vollständigkeit und wissenschaftliche Integrität der ganzheitlichen Studienauswertung sicherzustellen.

**Übermittlung Ihrer Daten außerhalb des Europäischen Wirtschaftsraums (EWR)**

Der Sponsor hat derzeit nicht die Absicht Ihre verschlüsselten Daten an Empfänger außerhalb der EU bzw. des EWR zu übermitteln. Sollten Ihre verschlüsselten Daten zukünftig an Empfänger außerhalb der EU bzw. des EWR übermittelt werden, geschieht dies ausschließlich für Zwecke, die der Erweiterung der Zulassung bzw. Zertifizierung des untersuchten Produkts oder zur Meldung von Unerwünschten Ereignissen^[[1]](#footnote-2)^, dienen.

Der Sponsor wird sich um Möglichkeiten bemühen, die Übermittlung im Rahmen eines der Mechanismen nach den Artikeln 45 (Angemessenheitsbeschlusses der EU Kommission) und 46 (Geeignete Garantien) DSGVO abzusichern, und die Ausnahmeregelungen nach Artikel 49 nur dann in Anspruch zu nehmen, wenn nicht auf diese Mechanismen zurückgegriffen werden kann.

**Wie lange und wo werden Ihre Daten aufbewahrt?**

Am Ende der Studie verbleibt eine Kopie Ihres digitalen Studienbogens (CRF) in der Klinik. Zusätzlich erhält die Klinik einen Export der Datenbank in elektronischer Form. Die elektronisch erfassten Daten werden nach Beendigung der Studie entsprechend den gesetzlichen Bestimmungen (mindestens 15 Jahre nach dem letzten Inverkehrbringen des Produktes) beim Sponsor (Aesculap AG) archiviert.

Nach der gesetzlichen vorgeschriebenen Aufbewahrungsfrist werden die Daten auf der Datenbank gelöscht. Ausgewertete Daten werden komplett anonymisiert, so dass dann keine weitere Re-Identifikation mehr möglich ist.

**Mit wem können Sie bei weiteren Fragen Kontakt aufnehmen?**

Wenn Sie Fragen zur Behandlung oder zu Ihrer Gesundheit haben, wenden Sie sich bitte an Ihren Studienarzt oder dessen Vertreter. Außerdem können Sie sich an den Datenschutzbeauftragten der Klinik wenden:

**Herrn Axel Peter**

Stabsstelle Recht

Doberaner Straße 142

18057 Rostock

Deutschland

Tel.: 0381 494 5155

E-Mail: datenschutz@med.uni-rostock.de

Sollten Sie Bedenken hinsichtlich der Erhebung und Verwendung Ihrer Daten haben, haben Sie das Recht, bei der für den Datenschutz zuständigen Aufsichtsbehörde der Klinik:

**Der Landesbeauftragte für Datenschutz und Informationsfreiheit Mecklenburg-Vorpommern,**

Werderstraße 74a, 19055 Schwerin

Telefon: +49 385 59494 0, E-Mail: info@datenschutz-mv.de

Telefax: +49 385 59494 58

sowie der zuständigen Aufsichtsbehörde des Sponsors:

**Landesdatenschutzbeauftragter Baden Württemberg**,

Postfach 10 29 32

70025 Stuttgart, Königstraße 10a, 70173 Stuttgart,

0711 / 61 55 41 – 0, poststelle@lfdi.bwl.de

Beschwerde einzulegen.

Die Ihnen hier vorgestellte Studie dient der Bestätigung der Leistungsfähigkeit eines zugelassenen Medizinproduktes. Sie als Patient tragen hierzu bei, wenn Sie sich für eine Teilnahme entscheiden. Hierbei entsteht Ihnen kein zusätzliches Risiko. Deshalb bedanken wir uns, dass Sie sich die Zeit genommen haben, eine Studienteilnahme in Betracht zu ziehen. Bitte richten Sie weitere Fragen direkt an den Studienarzt oder seine/en Vertreter.

Studienarzt:_______________________________________________________________________

Telefon: _________________________________________________________________________

Haben Sie weitere Fragen?

o Keine Fragen

o folgende Fragen sind noch offen:

Wir möchten Sie höflich um Erlaubnis bitten, Ihre Daten für diese Beobachtungsstudie zu verwenden.

**Vielen Dank für das Lesen dieser Informationen.**

## Example written informed consent form (German)

**E I N W I L L I G U N G S E R K L Ä R U N G**

**Prospektive, randomisierte, einfach verblindete Langzeitevaluation zum klinischen Ergebnis, dem Oxidationsprofil und der Abriebsanalyse bei moderat vernetztem Polyethylen mit oder ohne Vitamin E Zusatz beim künstlichen Kniegelenksersatz**

*Originaltitel: Prospective, randomized, single-blind, multinational, long-term study for the evaluation of the clinical outcome, oxidation profile and wear analysis of medium cross-linked Polyethylene with and without Vitamin E for total knee arthroplasty*

**Studienabkürzung: VIKEP**

Hiermit erkläre ich (Patient), dass ich am _________________ (Datum durch den Patienten eintragen) durch den Studienarzt bzw. seinem Vertreter, _________________________________ (Name des Arztes eintragen) über Ziele, Wesen und Bedeutung der oben genannten Beobachtungsstudie aufgeklärt wurde. Ich hatte ausreichend Gelegenheit, Fragen zu stellen und habe alle Punkte verstanden. Die Patienteninformation vom 02.07.2020 in der Version 1.0 habe ich sorgfältig gelesen und alle meine Fragen wurden zu meiner Zufriedenheit beantwortet. Ich hatte ausreichend Zeit, meine Entscheidung über die freiwillige Teilnahme an der Studie unbeeinflusst zu treffen.

**Datenschutz**

Ich bin mit der Erhebung, Speicherung und Nutzung meiner bei der Studie erhobenen personenbezogenen Daten gemäß den Angaben in der Patienteninformation (siehe Abschnitt „Was passiert mit Ihren Daten?“) einverstanden. Angaben über meine Gesundheit und die durchgeführte Therapie werden digital in dem webbasierten und elektronischen Datenerfassungssystem der Firma interActive Systems (“secuTrial“) sowie meine Röntgenbilder im Datenerfassungssystem des Zentrallabors der Raylytic GmbH („UNITY“) aufbewahrt und gespeichert. Alle dokumentierten Daten sind verschlüsselt (ohne Angabe von Namen, Initialen, Adressen oder Geburtsdaten) gespeichert. Die Daten werden zusammen mit einer fortlaufend generierten Nummer, meinem Alter/Geburtsjahr und Geschlecht abgespeichert. Meine Daten werden nach der Studie gemäß den gesetzlichen Bestimmungen (mindestens 15 Jahre nach dem letzten Inverkehrbringen des Produktes) beim Sponsor archiviert und anschließend gelöscht bzw. anonymisiert, (siehe Abschnitt „Wie lange und wo werden Ihre Daten aufbewahrt?“).

Mit meiner Unterschrift willige ich in die Weitergabe meiner verschlüsselten Daten an folgende Personen und Stellen ein:

1. an den Sponsor der Studie (Aesculap AG, Am Aesculap-Platz, 78532 Tuttlingen, Deutschland), die mit dem Sponsor verbundenen Unternehmen, wissenschaftliche Kooperationspartner (Raylytic GmbH, Schillerstr. 5, 04109 Leipzig) sowie an eine im Rahmen der Auftragsdatenverarbeitung verpflichtete Stelle.
2. an ggf. außerhalb der EU sitzende Stellen und verbundene Unternehmen des Sponsors mit einem geringeren Datenschutzniveau. Der Sponsor wird sich um Möglichkeiten bemühen, die Übermittlung im Rahmen eines der Mechanismen nach den Artikeln 45 (Angemessenheitsbeschlusses der EU Kommission) und 46 (Geeignete Garantien) DSGVO abzusichern, und die Ausnahmeregelungen nach Artikel 49 nur dann in Anspruch zu nehmen, wenn nicht auf diese Mechanismen zurückgegriffen werden kann.
3. an eine zur Verschwiegenheit verpflichtete vom Sponsor beauftragte Stelle oder eine Redaktion einer wissenschaftlichen Fachzeitschrift zum Zwecke der wissenschaftlichen Auswertung der Studiendaten und Veröffentlichung;
4. an zuständige Behörden oder Benannte Stellen in oder außerhalb der EU, wenn dies für Erweiterungen der Zulassung bzw. Zertifizierung des untersuchten Produkts erforderlich ist oder falls dies für die Meldung von Unerwünschten Ereignissen erforderlich ist.

Außerdem willige ich ein, dass eine vom Sponsor beauftragte und zur Verschwiegenheit verpflichtete Stelle/Person (Monitor) oder die zuständigen Behörden Einsicht in meine Daten und meine Patientenakte nehmen dürfen, soweit dies für die Überprüfung der ordnungsgemäßen Durchführung der Studie notwendig ist. Insoweit entbinde ich ausschließlich für diesen Zweck meine behandelnden Ärzte von der ärztlichen Schweigepflicht.

Meine Daten dürfen vom Sponsor oder von Personen, die vom Sponsor beauftragt wurden, für wissenschaftliche Auswertungen, Publikationen und Vorträge verwendet werden. Meine Anonymität wird dabei gewahrt.

Über meine Rechte im Zusammenhang mit der Verarbeitung meiner personenbezogenen Daten wurde ich informiert (siehe Abschnitt „Welche Rechte haben Sie in Bezug auf die Verarbeitung Ihrer Daten?“). Ich wurde auch darauf hingewiesen, dass meine Rechte auf Auskunft, Berichtigung und Einschränkung der Verarbeitung und Widerspruch eingeschränkt sein können, soweit es die Umsetzbarkeit der oben genannten Studie ernsthaft beeinträchtigen oder unmöglich machen würde.

**Von der Einwilligungserklärung und der Patienteninformation habe ich eine Kopie erhalten.**

Ich willige hiermit freiwillig in die Teilnahme an der Studie ein und weiß, dass ich diese Einwilligung jederzeit und ohne Angabe von Gründen widerrufen kann, ohne dass mir daraus Nachteile für meine jetzige oder zukünftige Behandlung entstehen.

Datum: I____I____I________I

TT MM JJJJ

__________________________________ _________________________

Name/Vorname des/der Patient/in Unterschrift

(**vom Studienteilnehmer eigenhändig einzutragen**)

**Bestätigung der Information des/der Patient/in durch den Studienarzt**

**Ich bestätige hiermit, den/die o.g. Patient/in ausführlich über Inhalt, Anliegen, Risiken und über die Freiwilligkeit der Teilnahme an der oben genannten Studie aufgeklärt zu haben.**

Datum: I____I____I________I

TT MM JJJJ

______ ____________________________________________________________________

Unterschrift des Studienarztes/Vertreter

_____________________________________________________________________________

Name und Funktion des Studienarztes (in Druckbuchsta

1. “Unerwünschtes Ereignis“: bezeichnet ein nachteiliges medizinisches Ereignis, eine nicht vorgesehene Erkrankung oder Verletzung oder nachteilige klinische Symptome, einschließlich anormaler Laborbefunde, bei Prüfungsteilnehmern, Anwendern oder anderen Personen im Rahmen einer klinischen Prüfung, auch wenn diese nicht mit dem Prüfprodukt zusammenhängen. [↑](#footnote-ref-2)
